# *Staphylococcus aureus* Toxins and Diabetic Foot Ulcers: Role in Pathogenesis and Interest in Diagnosis

**DOI:** 10.3390/toxins8070209

**Published:** 2016-07-07

**Authors:** Catherine Dunyach-Remy, Christelle Ngba Essebe, Albert Sotto, Jean-Philippe Lavigne

**Affiliations:** 1 Institut National de la Santé Et de la Recherche Médicale U1047, Université de Montpellier, UFR de Médecine, Nîmes 30908, France; catherine.remy@chu-nimes.fr (C.D.-R.); ngbachristelle@hotmail.fr (C.N.E.); albert.sotto@chu-nimes.fr (A.S.); 2 Service de Microbiologie, Centre Hospitalo-Universitaire Carémeau, Nîmes 30029, France; 3 Service des Maladies Infectieuses et Tropicales, Centre Hospitalo-Universitaire Carémeau, Nîmes 30029, France

**Keywords:** diabetic foot infection, *Staphylococcus aureus*, toxins

## Abstract

Infection of foot ulcers is a common, often severe and costly complication in diabetes. Diabetic foot infections (DFI) are mainly polymicrobial, and *Staphylococcus aureus* is the most frequent pathogen isolated. The numerous virulence factors and toxins produced by *S. aureus* during an infection are well characterized. However, some particular features could be observed in DFI. The aim of this review is to describe the role of *S. aureus* in DFI and the implication of its toxins in the establishment of the infection. Studies on this issue have helped to distinguish two *S. aureus* populations in DFI: toxinogenic *S. aureus* strains (harboring exfoliatin-, EDIN-, PVL- or TSST-encoding genes) and non-toxinogenic strains. Toxinogenic strains are often present in infections with a more severe grade and systemic impact, whereas non-toxinogenic strains seem to remain localized in deep structures and bone involving diabetic foot osteomyelitis. Testing the virulence profile of bacteria seems to be a promising way to predict the behavior of *S. aureus* in the chronic wounds.

## 1. Introduction

**Figure 1:**
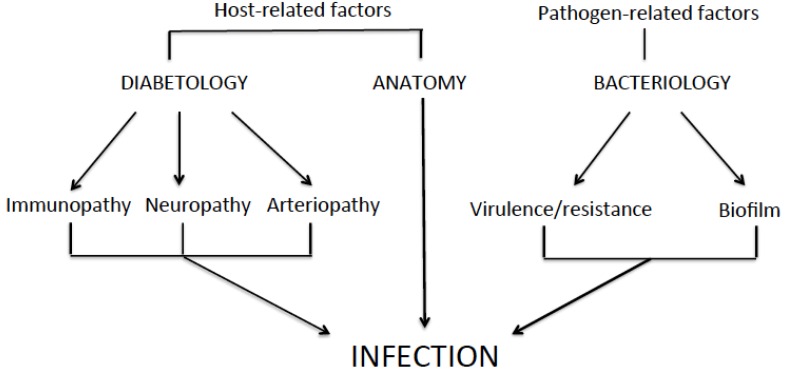
Interactions between metabolic, anatomical and bacteriological factors in diabetic foot infection.

Foot ulcers are common in diabetic patients. Its prevalence varies between 15% and 25% [1]. Infection of these ulcers is a frequent (40%–80%) complication representing a major cause of mortality and morbidity [2]. It is estimated to be the most common reason of lower-limb amputations [3,4,5]. The pathophysiology of diabetic foot infection (DFI) is quite complex. The prevalence and severity are a consequence of host-related processes (e.g., immunopathy, neuropathy and arteriopathy) and pathogen-related factors (e.g., virulence, antibiotic-resistance and microbial organization) (Figure 1) [6,7,8].

DFI pose many problems in clinical practice in terms of both management and diagnosis [9]. Indeed, the presence of impaired leukocyte functions and/or peripheral arterial disease may reduce the local inflammatory response and classical signs or symptoms of local infection [10,11]. Moreover, systemic signs of toxicity (e.g., leukocytosis or fever) may be lacking or appear late, even in severe cases [12,13,14]. Microbiological diagnosis of these DFI also encounters many limitations. As microorganisms colonize all chronic wounds, the diagnosis of DFI should not be based only on the microbiological analysis of a wound culture, but also on clinical findings [5,9,15]. The Infectious Diseases Society of America (IDSA) and the International Working Group on the Diabetic Foot (IWGDF) have developed clinical criteria for classifying the severity of DFI (Table 1) [15,16].

For many decades, culturing wound specimens were the only way to determine the causative pathogen(s) in a DFI. As microorganisms are always present on every skin wound and the DFI are often polymicrobial, the variability of bacterial virulence factors and the level of host resistance must also be taken into account. In fact, the different organisms isolated from infected wounds do not have a similar pathogenic impact, and evaluation of the intrinsic virulence potential of isolated bacteria to identify their real pathogenicity seems a promising way to best characterize the infection and to distinguish infection from colonization [16].

Several studies have shown that DFI are polymicrobial, and *Staphylococcus aureus* is the pathogen most frequently isolated [17,18,19,20,21,22]. *S. aureus* is both a commensal bacterium and a human pathogen. Indeed, approximately 30% of the human population is colonized with *S. aureus* [23]. Importantly, this bacterium causes a wide range of clinical infections (e.g., bacteremia, endocarditis, skin and soft tissue, osteoarticular, pulmonary and device-related infections) [24]. The numerous virulence factors and toxins produced by *S. aureus* during infection are well characterized [22]. However, some specific features could be observed in DFI. The aim of this review is to describe the role of *S. aureus* in DFI and the implication of its toxins in the establishment of the infection.

## 2. DFI and *Staphylococcus aureus*

### 2.1. Clinical Aspects of DFI

Many DFIs are superficial at presentation. However, bacteria can spread to subcutaneous tissues, including tendons, joints, fascia, muscle and bone. DFIs were classified by their clinical severity, ranging from mild (~35% of cases, depending on site of presentation), through moderate (~30%–60%), to severe (~5%–25%) [25]. The IWGDF and the IDSA have proposed simple clinical criteria for classifying the infection of diabetic foot ulcer (DFU) based on classical signs and symptoms of inflammation (Table 1) [3,15,25,26,27]. This scheme helps to predict whether hospitalization would be required and the clinical outcome. Moreover, various factors have been suggested as markers of DFI when classical signs are not obvious. These include the identification of friable or discolored granulation tissue, necrosis, fetid odor, non-purulent secretions, delay in healing despite otherwise adequate ulcer management and the discovery of unexplained hyperglycemia [16,25]. Interestingly, neither toxic shock syndrome nor toxinogenic manifestations could be clearly diagnosed in DFI.

### 2.2. Osteomyelitis

Infection of bone, or osteomyelitis, is found in ~50%–60% of patients hospitalized for a DFI and ~10%–20% of apparently less severe infections presenting in the ambulatory setting [28]. Bone infection results in inflammatory destruction, bone necrosis and new bone formation. It typically involves the forefoot and develops by contiguous spread from overlying soft tissue, penetration through the cortical bone and into the medullary cavity. The clinical presentation of diabetic foot osteomyelitis (DFOM) can vary with the site, the presence of any associated abscess or soft tissue involvement, the extent of bone infection, the bacterial species and the adequacy of limb perfusion. Osteomyelitis is frequently associated with vascular insufficiency [24,28,29].

Two clinical manifestations are frequently found during DFOM: an ulcer lying over a bony prominence (particularly when it fails to heal despite adequate off-loading) and a ‘sausage toe’. Osteomyelitis can, however, occur in the absence of overlying local signs of inflammation [30].

### 2.3. Matrix Metalloproteinases and DFI

During DFI, wound healing is hampered by mechanisms including a low growth factor activity, a reduced cellular proliferation, an elevated inflammatory markers and high levels of proteases [31].

The proteases are enzymes that act to control the degradation of extracellular matrix (ECM) [32]. The major group of proteases involved in the wound healing process are the matrix metalloproteinases (MMP) (e.g., MMP-2, MMP-8, MMP-9 and the serine proteases (human neutrophil elastase, HNE)). MMPs are endopeptidases whose physiological functions are to degrade the different components of the cutaneous tissue (collagen type I, elastin, etc.) and to facilitate keratinocyte migration and re-epithelialization [33,34]. HNE is an enzyme acting on a wide range of proteins in the ECM and on inflammatory mediators [31]. The MMPs’ activity is inhibited by tissue inhibitors of metalloproteinases (TIMPs) [35]. The balance between the level of proteases and their inhibitors is essential to allow a physiological healing process [36].

In DFI, under the hypoxic and inflammatory environment, the presence of elevated levels of MMPs could be noted as opposed to decreased levels of TIMPs [36,37,38,39]. This elevated protease activity participates in the important destruction of the ulcer ECM. It impairs the release of the different factors regulating the wound healing process (the collagen synthesis is deregulated; the growth factor synthesis and action are stopped) and affects extracellular matrix components, such as fibronectin [40]. All of these elements stall the wound in a chronic inflammatory phase without progressing to healing. However, no clear link between delayed healing and elevated protease activity has been described. This reinforces the need to understand the organization/cooperation between bacteria species that can modulate local inflammation and host MPP production.

In addition to human MMPs, some bacteria produce proteases that have a role in the healing of infected wounds. For example, the zinc-metalloproteinase, elastase, produced by *Pseudomonas aeruginosa*, induces degradation of fibroblast proteins and proteoglycans in chronic wounds and has also been shown to degrade host immune cell mediators. The microbial proteases participate also in the degradation of human ulcer fluid and inhibit fibroblast growth. It has now been suggested that human and bacterial MMPs act synergistically to maintain the lesion in a chronicity status [32]. Many pathogenic bacteria isolated on wounds (including *S. aureus*) are able to produce metalloproteinases.

### 2.4. Prevalence of S. aureus in DFIs

**Figure 2:**
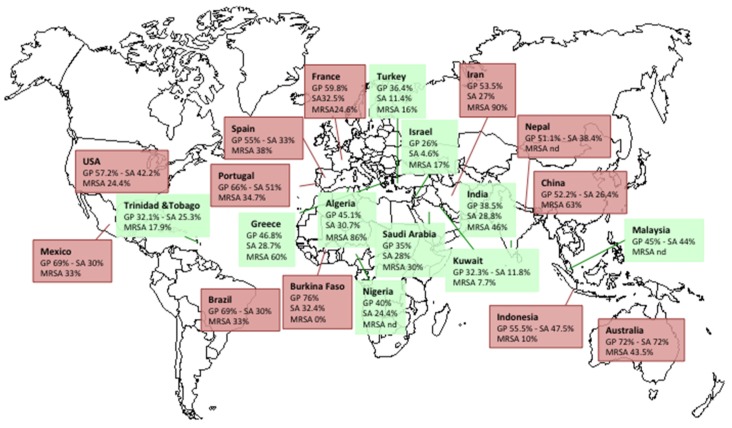
Worldwide geographic distribution of Gram-positive cocci (GP), *S. aureus* and Methicillin Resistant *S. aureus* (MRSA) isolated from diabetic foot ulcers. Red shading indicates regions where GP are predominant. Green shading indicates regions where Gram-negative bacilli are predominant.

In Occidental countries, Gram-positive aerobic cocci are the main microorganisms responsible for DFI with *S. aureus* the most commonly isolated bacteria, alone or in combination, in superficial or deep infection (Figure 2) [19,20,29,30,41,42,43,44,45,46,47,48,49,50,51,52,53,54,55,56,57,58,59,60,61,62,63,64,65,66,67,68,69,70,71,72]. In warmer countries (particularly in Asia and Africa), Gram-negative bacilli are more prevalent.

Many cases of deep infections and DFOM are polymicrobial. Also in this case, *S. aureus* is the main isolated bacteria, present in 30%–60% of cases [25].

### 2.5. Resistance of S. aureus in DFIs

The prevalence of methicillin-resistant *S. aureus* (MRSA) in DFI varies among countries with an exacerbation in countries that are less developed (Figure 2) [19,20,28,29,30,41,42,43,44,45,46,47,48,49,50,51,52,53,54,55,56,57,58,59,60,61,62,63,64,65,66,67,68,69,70,71,72,73]. MRSA are more often isolated from patients who have been previously hospitalized or reside in a chronic care facility, who have recently received antibiotic therapy or who have had a previous amputation [41,74].

In France, the prevalence of MRSA increased since the late 1990s [73,75]. Around 2005, different protocols were developed. National guidelines were implemented for the better management of DFI, notably concerning the debridement procedures, the microbiological samplings and antibiotic use [76]. The results of these guidelines entailed a significant decrease in the number of bacteria isolated per sample, in the increased rate of Gram-positive cocci and in the prevalence rate of multidrug-resistant bacteria, notably MRSA [3,15,28,77]. Concomitantly, hospital infection control measures have been improved [42,78,79], notably on the use of hydro-alcoholic solution for handwashing [80]. These measures have been associated with a reduction in MRSA diffusion [41].

For some authors, the isolation of MRSA in DFIs would be associated with more severe infections. However, different articles showed a similar clinical presentation and outcomes between MRSA and other pathogens [81,82,83].

Finally, some cases of DFIs due to vancomycin-resistant *S. aureus* have been described [84,85]. This type of resistance remains uncommon.

### 2.6. Pathogenesis

The pathogenesis of *S. aureus* in DFI is classical and corresponds to the physiopathology of skin and soft tissue infection (SSTI) [24,86]. The first defense against *S. aureus* infection is the neutrophil response. When *S. aureus* enters the injured skin, neutrophils and macrophages migrate to the site of infection. *S. aureus* evades this response using different methods (e.g., blocking sequestering host antibodies, chemotaxis of leukocytes, hiding from detection via capsule or biofilm formation and resisting destruction after ingestion by phagocytes).

**Figure 3:**
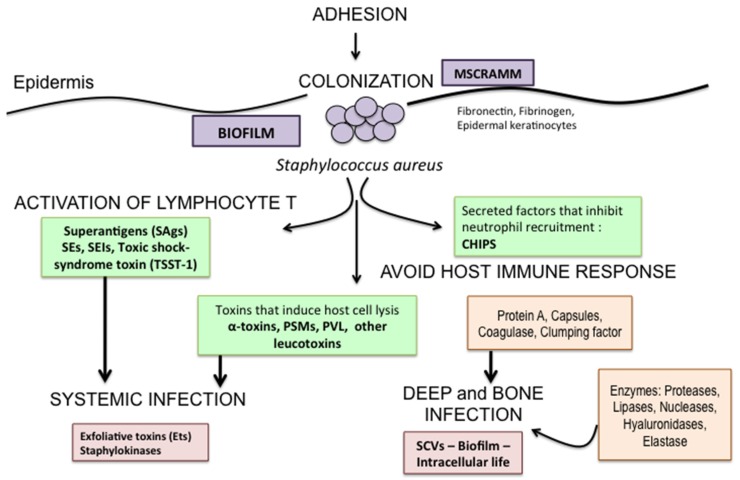
Infection process and virulence factors of *Staphylococcus aureus* in diabetic foot infection; MSCRAMM: microbial surface components recognizing adhesive matrix molecules. *S. aureus* secretes several virulence factors that evade host immune defenses. α-hemolysin (or α-toxin), phenol-soluble modulins (PSMs) and Panton-Valentine leukocidin (PVL) have the capacity to lyse host cells, which is a mechanism to evade immune responses. In addition, *S. aureus* secretes factors that inhibit neutrophil function, such as chemotaxis inhibitory protein of staphylococci (CHIPS). In addition, *S. aureus* possesses factors that active lymphocyte T cells: the superantigens (SE: enterotoxins; SEI: enterotoxin-like protein; TSST: toxic shock-syndrome toxin). On the other side, exfoliative toxins (Ets) facilitate bacterial skin invasion.

The knowledge of *S. aureus* pathogenicity reveals that these bacteria seem to be adapted for soft tissue and bone infections. Indeed the majority of infections remain localized to the feet. Generally, systemic infection secondary to diabetic foot is less prevalent (around 10%). This becomes particularly noticeable when analyzing the infection process [87]. The first event at the beginning of DFI is the adhesion to surface components (fibrinogen, fibronectin and epidermal keratinocytes). *S. aureus* attachment to ulcer surface depends on bacterial expression of numerous surface proteins that mediate adherence to components of bone matrix and collagen [88]. These bacterial cell surface receptors correspond to adhesins or microbial surface components recognizing adhesive matrix molecules (MSCRAMMs) [89,90,91,92] (Figure 3). MSCRAMMs facilitate bacterial adhesion to skin tissue. Moreover adhesins are essential in intracellular bone invasion. Indeed, *S. aureus* can invade osteoblasts [93], fibroblasts and endothelial cells. In the intracellular compartment this bacterium forms small-colony variants (SCVs) [94]. Thus, they are able to survive in a metabolically inactive state while preserving the integrity of the host cell. SCVs possess important metabolic and phenotypic differences from ordinary *S. aureus* isolates [94,95,96]. Indeed, they are relatively resistant to antibiotics [97,98] and, hence, difficult to eradicate with antibiotic therapy [99].

Moreover, the synthesis and secretion of glycocalyx play a role in the virulence of *S. aureus*. This is also documented for strains obtained from DFI [100]. The polysaccharide production begins immediately after the adhesion and covers the bacteria, representing an essential component for the development of a ‘biofilm’ (Figure 3) [101,102,103,104].

In addition to specific adherence mechanisms, *S. aureus* have a number of other virulence factors involved in the infection of soft tissues and bones. *S. aureus* is able to secrete toxins, which can lead to tissue necrosis (Figure 3). Toxins produced by *S. aureus* have an important role in the deepening and spread of the infection in the patient with DFI.

## 3. *Staphylococcus aureus* Toxins in DFIs

The ability of *S. aureus* to cause DFI is defined by numerous virulence factors among which secreted toxins play an important role (participation in colonization, persistence, evasion of the immune system and dissemination) [105]. These toxins include: the pore-forming toxins, the exfoliatins, the superantigen exotoxins (SAg) and the EDIN (epidermal cell differentiation inhibitors) toxins. These cytolytic toxins can damage membranes of host cells leading to cell lysis [106]. Hemolysins lyse red blood cells, while leukotoxins target white blood cells.

### 3.1. Pore-Forming Toxins

Pore-forming toxins (PFT) of *S. aureus*, through pore-forming and pro-inflammatory activities, have the ability to lyse host cells. They include the single-component α-toxin (or α-hemolysin), the phenol-soluble modulins (PSMs) and bi-component leukotoxins, including Panton-Valentine leukocidin (PVL), γ-hemolysin and leukocidin D/E [86].

#### 3.1.1. α-Toxin

This PFT is a beta-barrel forming toxin, which consists of beta sheets [107]. It is released by the majority of *S. aureus* as a water-soluble monomer [108]. Its targets are red blood cells and leukocytes except neutrophils [109]. Although α-toxin is the most frequently secreted, few studies have focused on the role of this hemolysin produced by *S. aureus* in DFI. In a French national study, almost all of the strains harbored the α-toxin-encoding gene *hla* independently of the grade [110]. However, this proportion varies between methicillin-susceptible *S. aureus* (MSSA) and MRSA. The α-hemolysin gene was significantly less present in MRSA (16.4%) than in MSSA strains (100%) [48]. As we noted previously, DFIs caused by MRSA present a similar severity of infections to MSSA, excluding an increased role of α-toxin in the pathogenicity of MSSA.

#### 3.1.2. Phenol Soluble Modulins

Recently, the role of a family of secreted peptides, the phenol-soluble modulins (PSMs), has been described in staphylococcal pathogenesis [111]. PSMs are produced by the majority of *S. aureus* strains [112]. These toxins are membrane-injuring toxins. They are structurally characterized as a family of seven small amphipathic α-helical peptides. Some PSMs are described: PSMα1–PSMα4 and delta-toxin. Like LukAB (described below), they induce human neutrophil lysis after phagocytosis, a pathogenesis mechanism of great importance for the high toxicity [113,114]. In DFI, to date, no report has evaluated the significance of these virulence factors in the pathogenicity of *S. aureus*.

#### 3.1.3. The Bi-Component Leukotoxins

*S. aureus* produces some bi-component toxins structurally similar to α-toxin. These toxins result from the association of the class S (Slow) component and the class F (Fast) component based on their electrophoretic mobility [105]. They induce the activation and the permeability of the target cells. They can lyse phagocytes (monocytes-macrophages and neutrophils), which is considered important for *S. aureus* immune evasion [115,116]. These PFTs include: (i) the gamma-toxin (gamma-hemolysins HlgA and HlgC/HlgB); (ii) the Panton-Valentine leukocidin (PVL) [117], corresponding to the LukS-PV and LukF-PV proteins; (iii) the leukocidins LukDE [118,119]; and LukAB [116] (also known as LukGH) [116].

The *hlg* gene cluster encoding for hemolysin-γ (Hlg) and hemolysin-γ2 (Hlg2) is located in the core genome. This cluster is present in almost all *S. aureus* strains. These toxins play a role in septic arthritis and could help community-acquired MRSA (CA-MRSA) to survive in human blood during infection [120,121]. In DFI, all of the isolated strains possess the different components of the *hlg* gene [48,110]. Interestingly, a variant of *hlg* (*hlgv*) is significantly associated with strains isolated from infected ulcers (Grades 2–4) [110,122].

The most well-known leukotoxin is PVL. PVL confers cytotoxicity on neutrophils and monocytes-macrophages, leading to a high virulence [123]. LukS-/LukF-PV are encoded within lysogenic phages [124,125]. Several *lukS-/lukF-PV*-transducing phages have been discovered [105]. This particular genetic organization involves an easy horizontal transmission of PVL genes in *Staphylococcus* spp. [126].

The PVL-positive strains are responsible for SSTIs (abscesses, furuncles, carbuncles or necrotizing fasciitis), severe necrotizing pneumonia and aggressive bone and joint infections [48,105,127,128]. However their prevalence is extremely diverse, varying between less than 5% and 67% of MSSA [129,130,131,132,133,134,135,136]. Interestingly, PVL-positive strains are statistically associated with younger patients [137]. This toxin has been linked to CA-MRSA infections [129], even if some CA-MRSA isolates do not carry the *lukS/lukF-PV* genes [138]. A high prevalence of CA-MRSA was observed in Africa [139], the Middle East [140,141], Asia [142] and America [143].

The PVL-producing clones were rarely isolated from DFI (Table 2) [48,110,122,144,145]. However, this prevalence varies between countries: France (~3%), Algeria and The Netherlands (~14%) [48,146]. The main PVL-producing strains isolated from DFI belong to ST80-MRSA (14/21 strains), followed by ST152 (6/21) and CC30 (1/21). ST80 is the main PVL clone circulating in Europe and North Africa [147,148,149]. Rates of PVL-producing clones in DFI remain low in comparison to data concerning SSTIs [127] (e.g., the epidemic furuncles (42%), major abscesses (73%) and gold surgically-drained abscesses (89%)) [146,150,151,152]. Indeed, in DFI, a higher incidence of PVL-positive isolates among subjects with CA-MRSA could be observed (31.8% versus 5.7%; *p* = 0.004) [153].

The role of PVL in DFI remains under debate. The different PVL clones are equally distributed among each grade [122]. The majority of Grade 1 ulcers where PVL-positive strains were isolated had a rapid amelioration [122], and for instance, in Algeria, all of the patients harboring PVL-positive strains isolated from DFI had a worsening evolution. However, the management of chronic wound infections in this country is clearly different from international recommendations, and the use of amputations is frequent, independent of the evolution of the wound. Finally, it is interesting to note that strains isolated from DFOM harbor neither *lukF-* nor *lukS-PV* [154]. Thus PVL-positive strains are scarce in wound ulcers, and their pathogenicity is not clearly established.

LukED exhibits toxicity toward PMNs in vitro and induces dermatonecrosis when purified toxin is injected into rabbits [118,119]. Moreover, this toxin plays a critical role in *S. aureus* lethality for mice. It targets and kills murine phagocytes (monocytes-macrophages and neutrophils), promoting disease progression [155]. In uninfected DFU, LukED is equally distributed among grades (52%–66%) when we pooled all of the data (Table 2). However, when data are analyzed separately, we showed that the *lukDE* gene was significantly more often associated with strains isolated from infected ulcers (Grades 2–4) [122]. In a more recent study, this gene was clearly identified as a marker that differentiated uninfected from infected ulcers and predicted the outcome of Grade 1 DFU [110]. The association between *lukDE* and MRSA has been also reported in DFI [48,156]. In DFOM, this gene was present in approximately 40% of strains [154]. However, even if experimental analysis showed the virulence potential of this leukotoxin, clinically, it seems to present a poorer activity compared to PVL, and the reduced virulence observed may be a response to atypical local inflammatory reaction [156].

The last leukocidin characterized is LukAB/HG. This new member of the *S. aureus* leukotoxin family contributes to neutrophil killing, promotes the survival of *S. aureus* in human whole blood, restricts neutrophil-mediated killing and promotes CA-MRSA pathogenesis [115,116]. No data concerning its implication in DFU have been reported.

### 3.2. Exfoliative Toxins

Exfoliative toxins are serine proteases secreted by *S. aureus*. Three (ETA, ETB and ETD) out of the four different serotypes of this toxin are linked to human infection. The exfoliatins act as “molecular scissors” facilitating bacterial skin invasion [157]. The prevalence of *eta* and/or *etb* ranges from 0.5%–3% in MSSA [157,158,159], whereas around 10% of MRSA are *eta*-positive [159].

In uninfected DFU, the distribution varies among clinical grades (Table 2). Interestingly, strains harboring these genes are three-times more frequent in Grade 4 (13.8%) as compared to Grade 1 (4%) or Grades 2–3 (3.5%). However, each exfoliatin does not have the same representation: if *eta* and *etb* are rare (1.3%) or absent, respectively, *etd* is the most prevalent (3.7%), particularly in strains isolated from Grade 4 (10.6%). In a previous study, we could also note that two of four patients harboring an *etd*-positive strain present on a Grade 1 ulcer had a worsening evolution [17]. Post et al. showed an important presence of *eta* (13%) and *etb* (17%) in DFI (no screening of *etD* was noted) [144]. However, as no grade has been reported in this work, it is not possible to link exfoliative genes and the severity of the DFI. Finally, this gene was absent in strains isolated from DFOM [154].

### 3.3. Enterotoxins

Enterotoxins are secreted toxins of ~20–30 kD that belong to the family of superantigens (SAg). These molecules over-induce cytokine production from both T-lymphocytes and macrophages [107]. The mechanisms by which staphylococcal enterotoxins work are not well known, but may include the activation of cytokine release, ultimately causing cell death by apoptosis. They contribute significantly to major illnesses [160,161]. A recent classification distinguishes three groups of SAg: staphylococcal enterotoxins (SEs), staphylococcal enterotoxin-like toxins (SEls) and toxic shock-syndrome toxin 1 (TSST-1) [162].

#### 3.3.1. Staphylococcal Enterotoxins and Enterotoxin-Like Toxins

The majority of *S. aureus* isolated from DFU have the capacity to produce a large number of Sags, notably SEs and SEls [161]. These toxins activate T cells, resulting in a high secretion of proinflammatory cytokines. This process leads to a chronic inflammatory state in uninfected DFUs, inducing a delay or an absence of wound healing [163]. Genes, including the *sea*, *sed*, *seg* and *sei* genes, code for enterotoxins found in *S. aureus* isolated from DFI. We observed that *sea* and *sei* are significantly more prevalent in Grade 2–4 ulcers than in Grade 1 (Table 2) [122] and could represent a biomarker to differentiate infection and colonization. The majority of enterotoxins are more frequently identified in MRSA strains except for *seb* and *seh* genes [122]. One of the main enterotoxins is SED, present in around 40% of the strains [122,161]. The *sed* gene is often located on a plasmid, and the active protein is structurally similar to SEA [164]. SEA could have a major role in atopic dermatitis by inducing the upregulation of adhesion molecules and eliciting inflammatory responses in endothelial cells and keratinocytes [165]. Thus, SED may be selected in DFU isolates because, similar to SEA, it has an enhanced ability to induce local inflammatory responses.

#### 3.3.2. Toxic Shock-Syndrome Toxin 1

The best known *S. aureus* superantigen is the 22-kD toxic shock-syndrome toxin 1 (TSST-1), which causes toxic shock syndrome (TSS). Additionally, SEl-X is a new member of the *S. aureus* SAg family, and it has been shown to have an important role in *S. aureus* necrotizing pneumonia infection caused by USA300 MRSA strain [166]. Vu et al. found that 88% of the DFU isolates carried the gene for SEl-X; the remainder contained the gene for TSST-1, and one isolate had genes for both SEl-X and TSST-1. Typically, *S. aureus* strains have the gene for either SEl-X or TSST-1 [161]. The prevalence of the *tsst* gene is low in the strains isolated from diabetic foot (~8%). However, as we observed for *etD*, this gene is more frequently present in Grade 4 (Table 2) and absent in DFOM [154].

### 3.4. Epidermal Cell Differentiation Inhibitors Toxins

EDINs toxins are members of a group of major bacterial virulence factors targeting host Rho GTPases [167]. Recent findings suggest that EDIN toxins might favor bacterial dissemination in tissues by a hematogenous route, through the induction of large transcellular tunnels in endothelial cells named macroapertures [168,169,170]. Indeed, recent data showed that EDIN toxins promote the formation of infection foci in a mouse model of bacteremia [171]. To date, three isoforms of EDIN have been characterized: the first discovered EDIN isoform (EDIN-A), isolated from the E-1 strain of *S. aureus* [172], as well as EDIN-B [173,174] and EDIN-C [175]. A first epidemiological survey, involving staphylococcal strains isolated from patients hospitalized for various infectious diseases, demonstrated a higher prevalence of *edin*-encoding genes in this group compared to nasal strains isolated from healthy patients [176]. Munro et al. showed that 90% of all *edin*-bearing *S. aureus* isolates carry the type-C allele. These isolates are more significantly associated with deep-seated soft tissue infections than other types of infections [177].

Messad et al. analyzed the distribution of *edin* genes in *S. aureus* isolated from DFI in a French national collection. *edin-B* is the most prevalent *edin* gene associated with DFIs (Table 2) [17]. The clonal complex analysis indicated that *edin*-positive strains belonged to four major groups: a singleton near CC8 (*edin-A*), a singleton belonging to ST152-MSSA (*edin-B*), CC80-MRSA (*edin-B*) and the most prevalent CC25/28-MSSA (*edin-B*). The distribution of *edin* genes in DFI shows an important presence of these genes in strains isolated from Grade 4 ulcers (2.5% for Grades 2–3 vs. 10.6% for Grade 4). Of note, patients with Grade 1 ulcers that presented *edin*-positive strains had a worsening evolution [17]. However, these genes seem to be absent in strains isolated from DFOM [154]. These observations support the idea that EDIN might work together with the arsenal of *S aureus* virulence factors to give the bacteria a higher potential for systemic infection [168]. *edin* encoding genes thus represent additional markers of interest to differentiate infecting from colonizing *S. aureus* strains in DFU and to predict the wound outcome.

## 4. Conclusions

In conclusion, DFI is a complex pathology involving the virulence of bacteria and host responses. Indeed, its main feature is the coexistence of multiple bacterial species on the chronic wound organized in pathogroups and the host-related responses encountered by bacteria, which modify the bacterial pathogenicity. Some studies showed the presence of toxinogenic *S. aureus* strains (harboring exfoliatins-, EDIN-, PVL- or TSST-encoding genes) in DFI, notably in Grade 4, with systemic impact. The absence of the same strains in DFOM suggests that these strains may not be adapted to colonize wounds. On the other hand, the main population of *S. aureus* (non-toxinogenic) isolated in uninfected DFU is perfectly adapted to infect deep structures and bone. The rarity of systemic cases (Grade 4) and of the presence of toxinogenic bacteria would, by definition, be an infected wound. However, it is interesting to note that some toxinogenic strains with a high pathogenic potential are present on uninfected wounds promoting no virulence in this polymicrobial environment. Moreover, no clear link between these strains and amputations has been noted to date. Subsequent studies are required to understand the role of toxins and their real impact in DFU. Screening the presence of genes encoding toxins by molecular biology tests on uninfected DFU could also represent a new approach for patients for whom the clinical diagnosis of infection is hampered by peripheral arterial disease, neuropathy or impaired leukocyte functions in the aim to predict if *S. aureus* is going to be invasive or not (needing an antibiotic treatment).

## Figures and Tables

**Table 1:** International Consensus on the Diabetic Foot classification of foot wound infections [3,15].

Grades	Symptoms
Grade 1	No symptoms, no signs of infection
Grade 2	Lesion only involving the skin (no subcutaneous tissue lesion or systemic disorders) with at least two of the following signs: - local warmth - erythema >0.5–2 cm around the ulcer - local tenderness or pain - local swelling or induration - purulent discharge (thick, opaque to white or sanguineous secretion) Other causes of inflammation of the skin must be eliminated (for example: trauma, gout, acute Charcot foot, fracture, thrombosis, venous stasis)
Grade 3	- Erythema >2 cm and one of the findings described above or - Infection involving structures beneath the skin and subcutaneous tissue, such as deep abscess, lymphangitis, osteomyelitis, septic arthritis or fasciitis There must not be any systemic inflammatory response (see Grade 4)
Grade 4	Regardless of the local infection, in the presence of systemic signs corresponding to at least two of the following characteristics: - temperature >39 °C or <36 °C - pulse >90 bpm - respiratory rate >0/min - PaCO_2_ <32 mmHg - leukocytes >12,000 or <4000/mm^3^ - 10% of immature leukocytes

**Table 2:** Repartition of toxin-encoding genes in *Staphylococcus aureus* isolated from diabetic foot ulcers at different clinical grades.

Genes	PEDIS Grades			Total	References
	Grade 1	Grades 2–3	Grade 4		
	*n* = 99	*n* = 481	*n* = 94	*n* = 674	
*lukF/luS-PV*	3 (3%)	15 (3.1%)	3 (3.2%)	21 (3.1%)	[48,110,122,145]
*edin*	6 (6.1%)	12 (2.5%)	10 (10.6%)	28 (4.1%)	[48,110,122,145]
*tsst*	5 (5.1%)	40 (8.3%)	12 (12.8%)	57 (8.5%)	[48,110,122,145]
*etA*, *etB*, *etD*	4 (4.0%)	17 (3.5%)	13 (13.8%)	34 (5.0%)	[48,110,122,145]
*sea*	39 (39.4%)	201 (41.8%)	39 (41.5%)	279 (41.4%)	[48,110,122,145]
*lukDE*	66 (66.7%)	263 (54.7%)	49 (52.1%)	378 (56.1%)	[48,110,122,145]
